# Mesenchymal Stem/Stromal Cell-Derived Extracellular Vesicles Elicit Better Preservation of the Intra-Renal Microvasculature Than Renal Revascularization in Pigs with Renovascular Disease

**DOI:** 10.3390/cells10040763

**Published:** 2021-03-31

**Authors:** Christopher M. Ferguson, Rahele A. Farahani, Xiang-Yang Zhu, Hui Tang, Kyra L. Jordan, Ishran M. Saadiq, Amir Lerman, Lilach O. Lerman, Alfonso Eirin

**Affiliations:** 1 Department of Internal Medicine, Division of Nephrology and Hypertension, Mayo Clinic, Rochester, MN 55901, USA; Ferguson.Christopher@mayo.edu (C.M.F.); Farahani.Rahele@mayo.edu (R.A.F.); zhu.xiangyang@mayo.edu (X.-Y.Z.); tang.hui@mayo.edu (H.T.); Jordan.Kyra@mayo.edu (K.L.J.); Saadiq.Ishran@mayo.edu (I.M.S.); lerman.lilach@mayo.edu (L.O.L.); 2 Department of Cardiovascular Diseases, Mayo Clinic, Rochester, MN 55901, USA; lerman.amir@mayo.edu

**Keywords:** renovascular disease, microvasculature, revascularization, mesenchymal stem/stromal cells, extracellular vesicles

## Abstract

Background: Percutaneous transluminal renal angioplasty (PTRA) confers clinical and mortality benefits in select ‘high-risk’ patients with renovascular disease (RVD). Intra-renal-delivered extracellular vesicles (EVs) released from mesenchymal stem/stromal cells (MSCs) protect the kidney in experimental RVD, but have not been compared side-by-side to clinically applied interventions, such as PTRA. We hypothesized that MSC-derived EVs can comparably protect the post-stenotic kidney via direct tissue effects. Methods: Five groups of pigs (*n* = 6 each) were studied after 16 weeks of RVD, RVD treated 4 weeks earlier with either PTRA or MSC-derived EVs, and normal controls. Single-kidney renal blood flow (RBF) and glomerular filtration rate (GFR) were assessed in vivo with multi-detector CT, and renal microvascular architecture (3D micro CT) and injury pathways ex vivo. Results: Despite sustained hypertension, EVs conferred greater improvement of intra-renal microvascular and peritubular capillary density compared to PTRA, associated with attenuation of renal inflammation, oxidative stress, and tubulo-interstitial fibrosis. Nevertheless, stenotic kidney RBF and GFR similarly rose in both PTRA- and EV-treated pigs compared RVD + Sham. mRNA sequencing reveled that EVs were enriched with pro-angiogenic, anti-inflammatory, and antioxidants genes. Conclusion: MSC-derived EVs elicit a better preservation of the stenotic kidney microvasculature and greater attenuation of renal injury and fibrosis compared to PTRA, possibly partly attributed to their cargo of vasculo-protective genes. Yet, both strategies similarly improve renal hemodynamics and function. These observations shed light on diverse mechanisms implicated in improvement of post-stenotic kidney function and position EVs as a promising therapeutic intervention in RVD.

## 1. Introduction

Atherosclerotic renovascular disease (RVD) remains an important cause of secondary hypertension and renal failure that affects almost 7% of individuals older than 65 years old [1]. Importantly, patients with RVD have a higher rate of cardiovascular complications, including myocardial infarction, congestive heart failure, flash pulmonary edema, or stroke, increasing all-cause mortality [2].

Several mechanisms are responsible for kidney injury distal to a stenosis, including oxidative stress, inflammation, defective angiogenesis, microvascular loss, and fibrosis [3]. Each of these injurious pathways contributes, to some degree, to functional deterioration in the post-stenotic kidney, and are likely to represent potential therapeutic targets to preserve the ischemic kidney in RVD.

Renin-angiotensin-aldosterone system (RAAS) blockade, statins, antiplatelet therapy, and lifestyle modifications are effective in achieving blood pressure control and minimizing cardiovascular risk in most RVD patients [4]. Percutaneous transluminal renal angioplasty (PTRA) supplemented with endovascular stenting does not provide additional benefit over conventional medical therapy with respect to prevention of clinical cardiovascular events in patients with RVD [5]. However, PTRA confers major clinical and mortality benefits in RVD patients presenting with flash pulmonary edema, rapidly declining kidney function, or refractory hypertension [6], underscoring the potential of restoration of renal artery patency to reduce risk for death in select ‘high-risk’ patients.

Emerging data suggest that novel treatment strategies that do not alter renal artery patency may also provide benefits in RVD. Our group has recently shown that intrarenal administration of adipose tissue-derived mesenchymal stem/stromal cells (MSCs) attenuates inflammatory injury and improves kidney function in patients with RVD [7]. The beneficial effects of MSCs are partly mediated by release of extracellular vesicles (EVs), which carry genetic and protein material capable of modulating angiogenesis, inflammation and oxidative stress in recipient cells. Furthermore, EVs carry pro-angiogenic genes capable of preserving the intra-renal microvasculature [8]. In line with this notion, we have previously shown that intra-renal delivery of EVs harvested from swine adipose tissue MSCs decrease inflammation, oxidative stress, and microvascular damage, and preserve the function of the stenotic pig kidney despite lingering renal artery stenosis and hypertension [8,9]. However, the efficacy of PTRA and MSC-derived EV-induced mitigation of intrarenal parenchymal damage and preservation of stenotic kidney function has not been directly compared.

The current study tested the hypothesis that MSC-derived EVs can comparably protect the post-stenotic kidney via their cargo of vasculo-protective genes.

## 2. Materials and Methods

Twenty-four female domestic pigs were studied after 16 weeks of observation with the approval of the Mayo Clinical Animal Care and Use Committee (A00003694-18). At baseline, animals were randomized in two groups: RVD (*n* = 18) and Controls (*n* = 6). RVD pigs were fed a high-cholesterol/high-carbohydrate diet (Purina Test Diet, Richmond, IN, USA) [10] and Controls a standard pig chow (Purina Animal Nutrition) for the duration of the study.

Six weeks later, all animals were anesthetized with 0.25 g of IM tiletamine hydrochloride/zolazepam hydrochloride (Telazol^®^, Fort Dodge Animal Health, New York, NY, USA) and 0.5 g of xylazine, and anesthesia maintained with 0.2 mg/kg/min of IV ketamine and 0.03 mg/kg/min of IV xylazine. Renal artery stenosis was induced endovascularly in RVD pigs, as previously described [11], whereas a sham procedure was performed in Control pigs. In RVD animals randomized to receive EVs, fat tissue was collected, and subsequently used to harvest autologous MSCs and their daughter EVs.

Six weeks after the induction of RVD or sham, all animals were similarly anesthetized and the degree of stenosis in each animal was determined by angiography. In 6 RVD pigs, PTRA was performed under fluoroscopic guidance as previously described [12], and technical success judged by repeating angiography 10–15 min (and 4 weeks) later. Additional 6 RVD pigs were treated with a single infusion of autologous MSC-derived EVs (1 × 10^11^, approximately 100 µg of protein) [8,9] into the stenotic kidney. The dose of EVs is supported by our previous studies in swine RVD which confirmed their safety and efficacy [8,9,13]. The remaining 6 RVD pigs and 6 Controls underwent only sham procedures consisting on renal angiography and saline infusion.

Four weeks later, all pigs were again similarly anesthetized and systemic blood samples collected to measure 8-isoprostane (enzyme immunoassay kit), plasma renin activity (PRA, GammaCoat kit; DiaSorin, Saluggia, Italy), and serum creatinine (spectrophotometry) levels [13]. In addition, a catheter was placed in the renal vein and to assess renin levels (ELISA, Sigma Aldrich, St. Louis, MO, USA, Cat# MAK157). Single-kidney hemodynamics and function were assessed in all pigs using multi-detector computed tomography (MDCT), and blood pressure measured with an intra-arterial catheter [14].

Three days after completion of MDCT studies, all animals were euthanized with an IV bolus of 100 mg/kg of sodium pentobarbital (Fatal-Plus, Vortech Pharmaceuticals, Dearborn, MI, USA). Stenotic kidneys were harvested, and sections frozen in liquid nitrogen (and maintained at −80 °C) or preserved in formalin for ex vivo studies. In addition, a lobe of kidney tissue was perfused and prepared for micro-CT studies.

### 2.1. In Vivo Studies

Single-kidney hemodynamics and function were measured using a Flash 128 MDCT scanner (Somatom Definition Flash, Siemens Healthcare, Newark, DE, USA), as previously described [14,15]. Several multi-scan exposures (cycle time = 0.67 s) were acquired after a bolus of iopamidol (0.5 cc/kg over 2 s), followed by additional 70 scans (cycle time = 2 s). Volume images were reconstructed and regions of interest traced from cross-sectional images from the aorta, renal cortex, and medulla, which generated average tissue attenuation curves. These curves were then fitted to calculate (Analyze, Biomedical Imaging Resource, Mayo Clinic, Rochester, MN, USA) single-kidney glomerular filtration rate (GFR, cortical curve slope) and renal blood flow (RBF) factored to body weight, as described [16,17].

### 2.2. Ex Vivo Studies

MSC and EV isolation, characterization, and culture.

MSCs were harvested from abdominal adipose tissue from 6 RVD pigs six weeks after baseline. Cells were cultured in advanced minimal essential medium (Gibco/Invitrogen, Carlsbad, CA, USA) with platelet lysate (Mill Creek Life Sciences, LLC, Rochester, MN, USA) [18,19,20,21], kept in cell recovery medium, and characterized by the expression of CD44+, CD90+, and CD105+ [18,22], and differentiation into adipocytes, osteocytes, and chondrocytes [18,19,20,21]. EVs were then isolated from supernatants of MSCs (10 × 10^6^) by ultracentrifugation [18], and characterized by the expression of MSC (CD44+, CD90+, and CD105+) and EV (CD63+, CD9+, and CD81+) markers [19,23,24], as we have previously described. EVs and MSCs were then stored at −80 °C to study their mRNA cargo, as described below. In addition, EVs were subsequently labeled with a red fluorescence dye (PKH26, Sigma) and injected into the stenotic kidney of 6 RVD pigs.

### 2.3. EV mRNA Cargo

High throughput mRNA sequencing (mRNA-seq) was performed to compare the expression of vasculo-protective genes between MSCs and their EVs. We have previously shown that the cargo of MSC-derived EVs comprises a selective set of mRNAs rather than random membrane-encased genes [18]. We characterized and compared the expression profile of pro-angiogeneic, anti-inflammatory, and antioxidant mRNAs between EVs and their parent MSCs to identify genes selectively enriched in EVs. mRNA-seq libraries were prepared using the TruSeq RNA Sample Prep Kit (Illumina, San Diego, CA, USA), MSCs and EVs were sequenced, and data analyzed with the MAPRSeq system, TopHat [23,24] and featureCounts [25], as previously described [18]. mRNA data was normalized and expressed as reads per kilobasepair per million mapped reads. Pro-angiogenic, anti-inflammatory, and antioxidants genes were identified with GeneCards^®^ database (http://www.genecards.org/, accessed on 2 March 2021), and genes with RPKM > 0.1, fold-change (EVs/MSCs) > 1.4, and *p* values < 0.05 (EVs vs. MSCs, Student’s *t*-test) were classified as enriched in EVs. Search Tool for the Retrieval of Interacting Genes (STRING) version 9.1 (http://string-db.org/, accessed on 4 February 2021) was used to predict associations among genes enriched in EVs.

### 2.4. EV Tracking

EV retention and localization were explored in stenotic kidney sections, as previously described [8,9]. Labeled (PKH26) EVs in renal sections were counted manually under fluorescence microscopy and the total cross-sectional area calculated using ZEN^®^ (Carl ZEISS SMT; Oberkochen, Germany). The average number of EVs/square millimeter was multiplied by the section thickness and by the total renal volume, which was obtained by MDCT. EV retention rate (%) was calculated by dividing the total number of EVs/kidney by the number of injected EVs. EV localization was evaluated in stenotic kidney sections stained with the tubular marker phaseolus vulgaris erythroagglutinin (PHA-E, Vector Lab, Cat# FL-1121, 1:500) [9], and the endothelial marker CD31 (Abcam, Cambridge, UK, Cat# ab28364, 1:50) [8].

### 2.5. Renal Microvascular Density

Renal microvascular architecture was assessed using 3D micro CT, and images analyzed as previously described [26]. Kidneys were perfused with an intravascular radio-opaque silicone polymer (Microfil MV122, Flow Tech, Carver, MA, USA) under physiological pressure using a syringe infusion pump (SIP 22; Harvard Apparatus, Holliston, MA, USA) through a cannula (PE 190) ligated in a segmental renal artery. Perfused sections from the cortex and medulla were preserved in 10% buffered formalin, and subsequently prepared and scanned using a micro-CT scanner. Spatial density (number of vessels/tissue area) of cortical microvessels (diameters of 20–500 μm) and microvascular tortuosity (an index of vessel immaturity) were calculated using Analyze™. In addition, peritubular capillaries were counted in Hematoxylin and Eosin-stained slides at ×100, and expressed as number of capillaries per tubules [27], whereas renal expression of the pro-angiogenic factor vascular endothelial growth factor (VEGF) was measured by Western blotting (sc-152, Santa Cruz, 1:200) [28].

### 2.6. Renal Injury

In situ renal production of superoxide anion was assessed by immunofluorescence microscopy using dihydroethidium (DHE) [12], and intra-renal inflammation by interleukin (IL)-6 immunoreactivity (Abcam Cat# ab6672 1:400). Tubular injury was scored in sections stained with Periodic acid-Schiff (PAS) [28,29], whereas tubulo-interstitial fibrosis (area %) and glomerulosclerosis (% of sclerotic out of 100 glomeruli) were assessed in Trichrome-stained slides [30].

### 2.7. Statistical Analysis

All statistical tests were performed using JMP Pro version 14 (SAS) software. Normally distributed data were expressed as mean ± SD and non-normally distributed data as median range. Parametric (ANOVA and 2-tailed Student’s *t*-test) and nonparametric (Wilcoxon and Kruskal–Wallis) methods were used as appropriate. Results were considered significant if *p* < 0.05.

### 2.8. In Vitro Studies

EV pro-angiogenic effects were assessed by their capacity to induce in human umbilical endothelial cells (HUVECs, PromoCell, Heidelberg, Germany) to migrate and form tube-like networks on matrigel [31,32]. These functional attributes were assessed in HUVECs incubated alone, or in combination with MSC-derived EVs. HUVEC migration was measured using a Boyden Chamber assay (Millipore 5 µm QCM Chemotaxis Cell Migration Assay, Millipore Sigma, Burlington, MA, USA, cat#: ECM506) [33]. The Boyden Chamber system uses a hollow plastic chamber, sealed at one end with a porous membrane. Cells were placed inside the 24-well-colorimetric chamber and allowed to migrate through the pores to the other side of the membrane. Migratory cells were then stained by crystal violet and quantified by spectrophotometry at an optical density of 560 nm (SynergyMx, BioTek Instruments Inc., Winooski, VT, USA).

Tube formation was assessed as previously described [31]. Matrigel (BD Biosciences, Bedford, MA, USA) was spread onto 24-well plates (Coster, Corning Inc., Corning, NY, USA) and allowed to polymerize for 15 min at 37 °C. HUVECs (4 × 10^4^) were plated on matrigel precoated well plates and incubated at 37 °C for 24 h with EGM-2 culture medium. Tube length and number were counted in 4 random (×20) fields per subject and measured using Image-J (Version 1.5, National Institute of Health) [34]. Experiments were done in triplicate and observers blinded to cell type and group.

In addition, we measured the expression of the anti-inflammatory cytokine interleukin (IL)-13 (ELISA, ThermoFisher, Waltham, MA, USA, catalog# is ESIL13) in pig proximal kidney tubular epithelial cells (LLC-PK1, ATCC, Manassas, VA, USA) untreated or treated with MSC-derived EVs.

## 3. Results

All RVD pigs achieved comparable hemodynamically significant stenosis and similar levels of hypertension immediately before sham or therapy with PTRA or MSC-derived EVs (all *p* < 0.05 vs. Control, *p* > 0.05 ANOVA among RVD groups). At the end of the study, body weight was similarly elevated in all RVD groups compared to Control (Table 1). PTRA fully restored renal artery patency. Systolic, diastolic, and mean arterial pressure that increased in RVD + Sham compared to Control decreased in RVD + PTRA, but remained elevated in RVD + EV pigs (*p* = 0.20 vs. RVD + Sham). Circulating isoprostane levels were similarly elevated in RVD + Sham and RVD + PTRA compared to Control, but decreased in RVD pigs treated with EVs. Renal vein renin levels were higher in all RVD compared to Control, but decreased only in PTRA-treated pigs, whereas systemic PRA levels were similar among all groups. Serum creatinine levels that were higher in RVD compared to Control, equally decreased in PTRA- and EV-treated pigs. Likewise, single-kidney RBF and GFR similarly increased in RVD + PTRA and RVD + EVs versus RVD + Sham.

### 3.1. EVs Were Retained in the Stenotic Kidney

**Figure 1:**
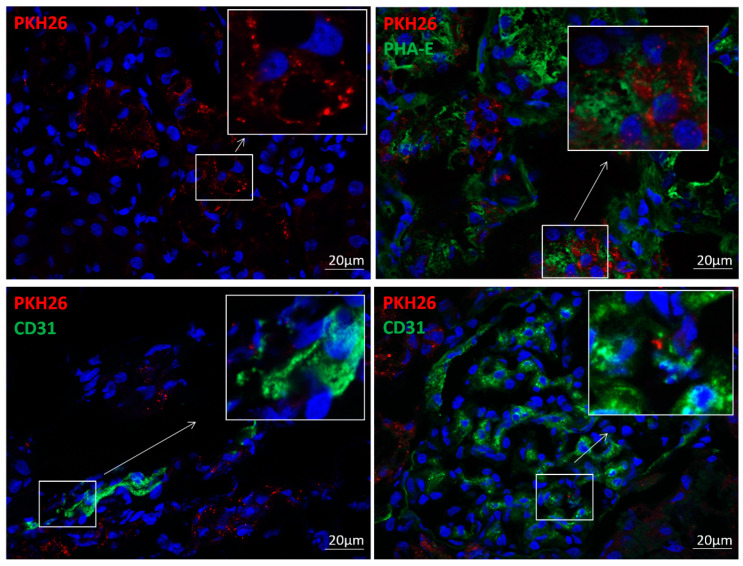
MSC-derived EVs were retained in the post-stenotic kidney. EV clusters (PKH26, red) were detected in the swine stenotic kidney 4 weeks after intra-renal delivery (upper left). Immunofluorescence co-staining with phaseolus vulgaris erythroagglutinin (PHA-E, upper right) and CD31 (green, bottom), suggesting EV engraftment in renal tubular cells and endothelial (peritubular capillary and glomerular) cells, respectively.

Four weeks after intra-arterial administration, 2–3% of injected EVs were retained in the stenotic kidney, reflected in immunofluorescence staining by the presence of EV clusters in the tubulo-interstitium of EV-treated kidneys (Figure 1). Some EVs co-localized with PHA-E+ and CD31+ cells, suggesting EV engraftment in tubular cells and endothelial cells.

### 3.2. EVs Exerted Greater Improvement in the Intra-Renal Microvasculature Than PTRA

**Figure 2:**
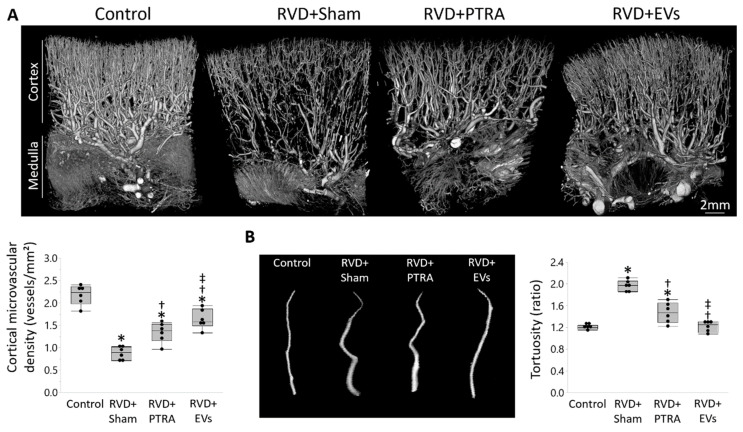
EVs exerted greater improvement in the stenotic kidney microvasculature than PTRA. (**A**) Representative 3D micro-computed tomography images of the pig kidney and quantification of spatial density of cortical microvessels that decreased in RVD + Sham compared to Control, increased in RVD + PTRA, and further increased in EV-treated pigs. (**B**) Microvascular tortuosity that increased in RVD + Sham versus Control, decreased in RVD + PTRA, but was restored to normal levels in EV-treated pigs. * *p* < 0.05 vs. Control, ^†^ *p* < 0.05 vs. RVD + Sham, ^‡^ *p* < 0.05 vs. RVD + PTRA.

**Figure 3:**
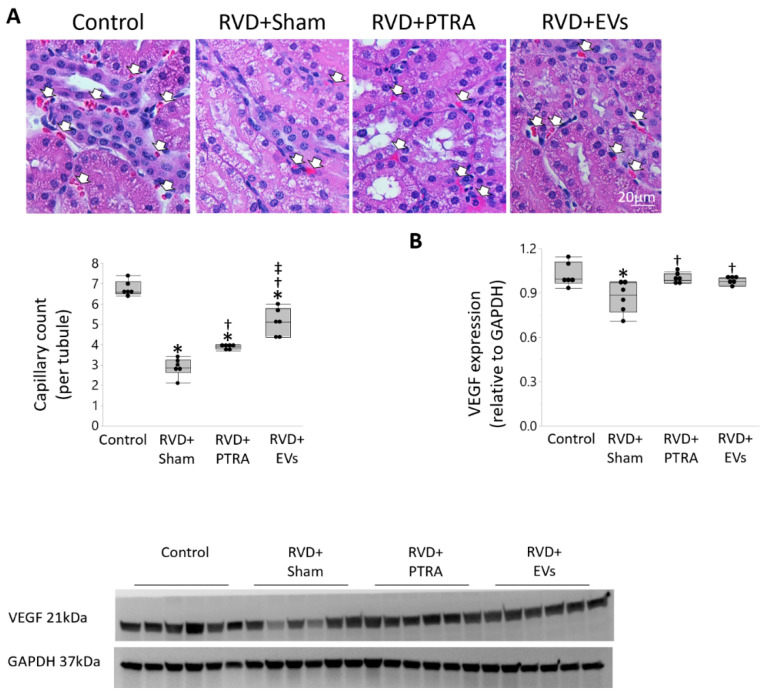
EVs elicited superior improvement in stenotic kidney peritubular capillary density than PTRA. (**A**) Kidney hematoxylin and eosin (H&E) staining and quantification of peritubular capillary density (arrows), which decreased in RVD + Sham compared to Control, increased in RVD + PTRA, and further increased in RVD + EV pigs. (**B**) Renal expression of VEGF that decreased in RVD + Sham compared to Controls, similarly improved in both PTRA- and EV-treated pigs. * *p* < 0.05 vs. Control, ^†^ *p* < 0.05 vs. RVD + Sham, ^‡^ *p* < 0.05 vs. RVD + PTRA.

Spatial density of cortical microvessels markedly decreased in RVD + Sham pigs compared to Controls, increased in RVD + PTRA, but further increased in RVD + EVs (Figure 2A). Microvascular tortuosity was substantially higher in RVD + Sham versus Controls, decreased in RVD + PTRA, but further decreased in EV-treated pigs (Figure 2B). Similarly, peritubular capillary density that decreased in RVD + Sham slightly increased in RVD + PTRA, and further increased in RVD + EV pigs (Figure 3A). However, renal expression of VEGF that decreased in RVD + Sham compared to Controls, similarly improved in both PTRA- and EV-treated pigs (Figure 3B).

### 3.3. EVs Produced More Attenuation of Renal Injury Compared to PTRA

**Figure 4:**
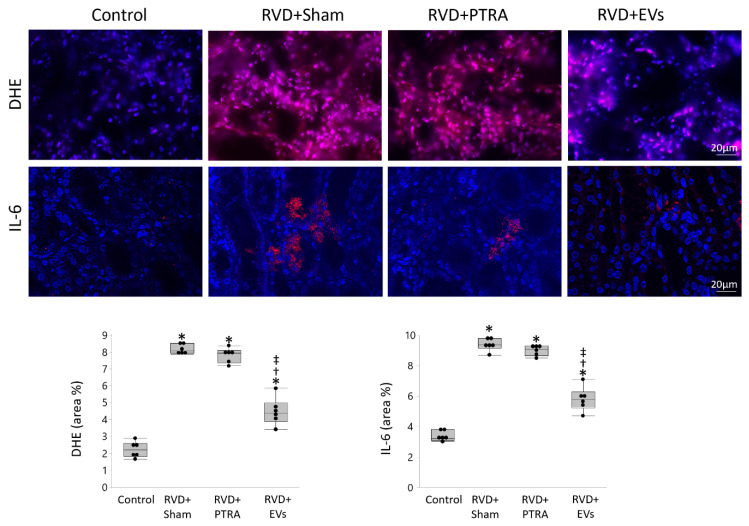
PTRA failed to decrease oxidative stress and inflammation in the stenotic kidney. Representative kidney dihydroethidium (DHE, red) and interleukin (IL)-6 (red) staining, and their quantification (area %) showing that in situ production of superoxide anion and IL-6 immunoreactivity similarly increased in RVD + Sham and RVD + PTRA compared to Control, but decreased in EV-treated pigs. * *p* < 0.05 vs. Control, ^†^ *p* < 0.05 vs. RVD + Sham, ^‡^ *p* < 0.05 vs. RVD + PTRA.

**Figure 5:**
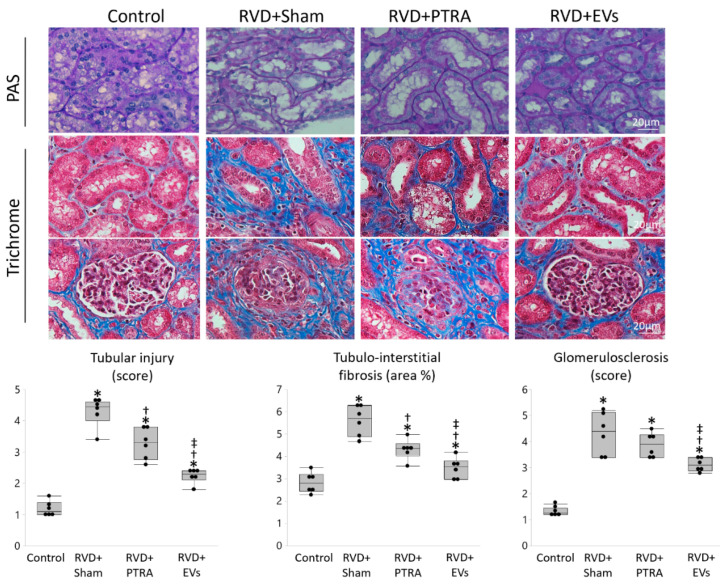
EVs produced more attenuation of renal tubular injury and fibrosis than PTRA. Representative Periodic acid-Schiff (PAS) and Trichrome staining and quantification of tubular injury score, tubulo-interstitial fibrosis, and glomerulosclerosis. Tubular injury and tubulo-interstitial fibrosis were higher in RVD + Sham versus Control, decreased in RVD + PTA, but further decreased in EV-treated pigs. Glomerulosclerosis similarly increased in RVD + Sham and RVD + PTRA compared to Control, but decreased in RVD + EVs. * *p* < 0.05 vs. Control, ^†^ *p* < 0.05 vs. RVD + Sham, ^‡^ *p* < 0.05 vs. RVD + PTRA.

Renal superoxide production increased in RVD + Sham compared to Control, remained unchanged by PTRA, but decreased in EV-treated pigs, as did IL-6 immunoreactivity (Figure 4). Tubular injury score was higher in RVD + Sham compared to Control, moderately decreased in PTRA-treated pigs, but significantly decreased RVD + EVs pigs, as did tubulo-interstitial fibrosis (Figure 5). Contrarily, glomerulosclerosis that similarly increased in RVD + Sham and RVD + PTRA compared to Control, decreased only in EV-treated pigs.

### 3.4. MSC-Derived EVs Were Enriched with Vasculo-Protective Genes

**Figure 6:**
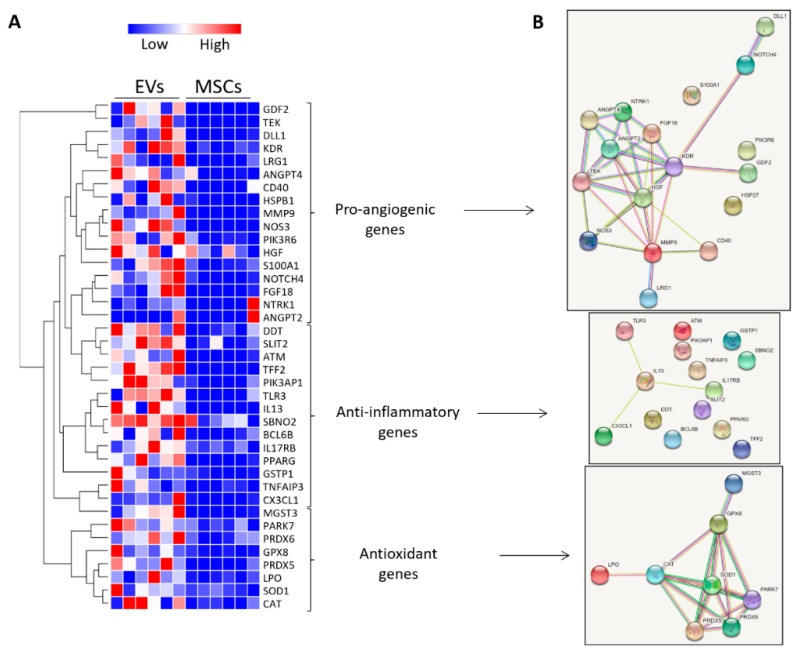
MSC-derived EVs are enriched with vasculo-protective genes. (**A**) Heat map of pro-angiogeneic, anti-inflammatory, and antioxidant mRNAs upregulated in EVs compared to their parent MSCs (fold change > 2, *p* < 0.05). (**B**) Interaction network (STRING) of genes enriched in EVs. Nodes represent mRNAs and color lines their interactions according to the functional association networks from the STRING database.

To explore potential mechanisms by which EVs elicited better preservation of the intra-renal microvasculature than PTRA, we compared the genetic cargo of vasculo-protective genes between EVs and their parent MSCs using mRNA-seq (See Supplementary Materials). mRNA-seq identified that 17 of 121 pro-angiogenic genes, 14 of 228 anti-inflammatory genes, and 8 of 48 antioxidant genes were enriched in EVs versus MSCs (Figure 6A). STRING analysis revealed several known or predicted interactions among these genes and identified a range of interacting groups around the pro-angiogenic kinase insert domain receptor and hepatocyte growth factor (HGF), the anti-inflammatory IL-13, and the antioxidant superoxide dismutase (SOD)-1 genes (Figure 6B).

### 3.5. EVs Modulate Angiogenesis and Inflammation in Recipient Cells

**Figure 7:**
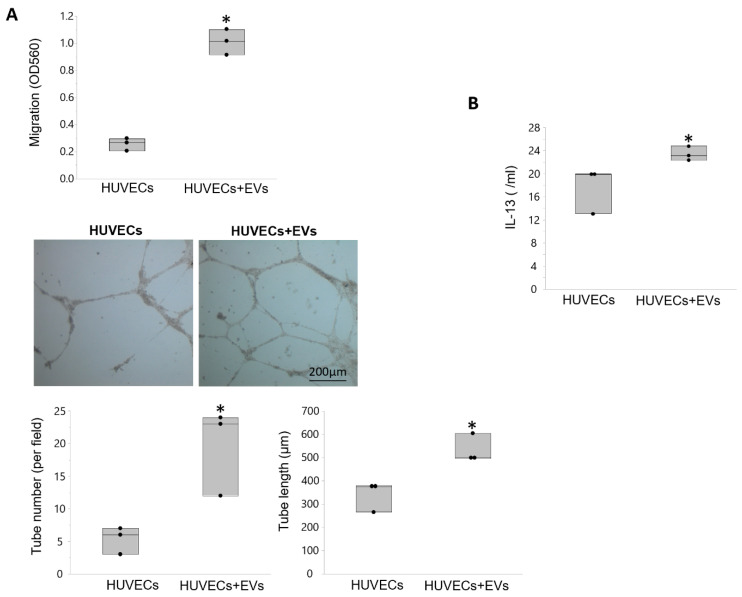
EVs modulate angiogenesis and inflammation in vitro. (**A**) Human umbilical endothelial cells (HUVEC) migration increased in cells treated with EVs compared to untreated HUVECs, as were the number and length of tubular-like structures formed in matrigel. (**B**) Treatment of swine renal tubular (PK1) cells with EVs was associated with increased expression of the anti-inflammatory cytokine IL-13. * *p* < 0.05 vs. HUVECs.

Cell migration was higher in HUVECs treated with EVs versus untreated HUVECs, as were the number and length of tube-like structures on Matrigel (Figure 7A). Transfection of PK1 cells with EVs increased expression of the anti-inflammatory cytokine IL-13 (Figure 7B).

## 4. Discussion

The current study compared the efficacy of two clinically applicable approaches for modifying post-stenotic kidney injury and dysfunction in experimental RVD. Specifically, we compared the reno-protective properties of technically successful PTRA that restores renal artery patency and decreases blood pressure with the effects of intrarenal delivery of MSC-derived EVs that does neither, but improves the renal parenchyma directly. We found that a single intrarenal delivery of MSC-derived EVs elicited a greater attenuation of intrarenal parenchymal injury compared to renal revascularization. EVs exerted greater improvement in stenotic kidney cortical microvascular and peritubular capillary density compared to PTRA, and improved vessel maturity, possibly partly attributed to their cargo of vasculo-protective genes. Importantly, EV-induced preservation of the stenotic kidney microvasculature was associated with amelioration of oxidative stress and inflammation, and more attenuation of tubular injury and fibrosis than PTRA. Nevertheless, both interventions achieved similar improvement of renal hemodynamics and function, suggesting that moderate attenuation of intrarenal parenchymal damage is enough to achieve short-term improvements of renal function in experimental RVD.

Despite disappointing results from prospective randomized trials [5], restoring renal artery patency is effective in some individuals with RVD. Clinical and mortality benefits significantly improve in select ‘high-risk’ RVD patients following renal revascularization [6]. However, PTRA may induce greater deterioration of renal function, possibly secondary to peri-procedural complications [35], limiting its clinical value in unselected cohorts.

Recent clinical and experimental data suggest a role for stem/stromal cell-based therapy with MSCs to preserve the structure and function beyond a stenotic lesion [7,22]. Similarly, intrarenal delivery of MSC-derived EVs attenuates stenotic kidney injury and dysfunction in experimental RVD without affecting renal artery stenosis or blood pressure levels [8,9]. The beneficial effects of EVs have been partly attributed to their ability to ameliorate intrarenal microvascular rarefaction, an important determinant of the progression of renal injury in RVD [12,36]. In line with this, this study shows that EVs elicited greater preservation of cortical microvascular density and peritubular capillary density compared to PTRA. Furthermore, MSC-derived EVs showed superior attenuation of stenotic kidney microvascular remodeling, reflected in lower vessel tortuosity. Tortuous microvessels are unstable and hyperpermeable [37], thereby the more prominent decrease in vessel tortuosity in EV-treated pigs reflects greater gains in vascular maturity.

It is undoubted that angiogenic competence promotes renal microvascular recovery. Neovascularization encompasses several concatenated events, including endothelial cell proliferation, migration, differentiation, and extracellular matrix remodeling, which are promoted by pro-angiogenic factors [38]. Indeed, intrarenal delivery of the pro-angiogenic VEGF [39] or HGF [40] preserves the stenotic kidney microcirculation in experimental RVD. The current study shows that swine MSC-derived EVs are enriched with several pro-angiogenic genes, including HGF and KDR, which encodes one of the receptors of VEGF. In addition, EVs contained members of the angiopoietin and NOTCH signaling pathways, which operate in concert to promote vascular development and maturation of newly formed vessels, ultimately fostering microvascular stabilization [41,42]. Therefore, delivery of EVs and subsequent engraftment in peritubular capillary endothelial cells could have contributed partly to microvascular protection of EV-treated pigs. Interestingly, renal expression of VEGF was similarly restored in EV- and PTRA-treated pigs, implying that additional angiogenenic mechanisms contributed to the greater microvascular protection of EVs versus PTRA.

Amelioration of renal inflammation and oxidative stress might have accounted partly for the superior microvascular-protective effect of EVs compared to PTRA. EVs were enriched with several genes capable of modulating renal inflammation. Intrarenal microvessels are susceptible to cytokines released by inflammatory cells infiltrating the renal parenchyma, which compromise the integrity and function of endothelial cells [43]. Thus, EVs that were retained in the tubulo-interstitium and subsequently released their anti-inflammatory cargo could have contributed to preserve microvascular architecture beyond the stenotic lesion. Among anti-inflammatory genes packed in EVs are IL13 and IL17, which promote polarization of macrophages to an M2 reparative phenotype [44,45]. Microvascular rarefaction can also promote activation of inflammatory cytokines, creating a vicious cycle of renal inflammation and microvascular damage. In line with this, we found that immunoreactivity of the pro-inflammatory IL-6 decreased in EV-treated pigs, implying that EV-induced preservation of the microvasculature could have also contributed to ameliorate inflammation in the stenotic kidney. In contrast, renal expression of IL-6 remained unaltered in PTRA-treated pigs, in line with our previous observation of sustained renal release of inflammatory biomarkers following renal revascularization in human RVD [46,47].

EVs were also enriched with several genes encoding for important antioxidant proteins, including SOD-1, catalase, and peroxiredoxins. Reactive oxygen species increase vascular tone, sensitivity to vasoconstrictors, and endothelial dysfunction, contributing to cortical microvascular loss in the post-stenotic kidney [48]. Therefore, EVs bearing antioxidant properties might have thereby ameliorated both systemic and renal oxidative stress, reflected in decreseased circulating isoprostanes and renal superoxide anion production. Contrarily, PTRA failed to reduce systemic and renal oxidative stress, in agreement with our previous findings in swine RVD [12,22]. Therefore, despite similar improvement of renal angiogenes, the anti-inflammatory and antioxidant effects of EVs likely contributed to better preserve the number and maturity of newly formed microvessels compared to PTRA.

Importantly, EV-induced microvascular protection was also associated with greater attenuation of renal tubular damage and tubulo-interstitial fibrosis. Furthermore, our in vitro studies showed that EVs increase HUVEC migration, as well as the number and length of tubes compared to untreated HUVECs. Likewise, treatment of swine renal tubular cells with EVs was associated with increased expression of the anti-inflammatory cytokine IL13, underscoring the potential of EVs to modulate angiogenesis and inflammation in recipient cells. Possibly, the combined anti-inflammatory, antioxidant, and vasculo-protective effects of MSC-derived EVs might have ameliorated renal injury, fibrosis, and glomerulosclerosis in the post-ischemic kidney. It is not unlikely that persistence of these mechanisms might have curtailed renal structural recovery 4 weeks after PTRA. Therefore, adjunctive measures need to be implemented during or after endovascular stenting to modify these pathways and preserve the structure of the post-ischemic kidney.

Interestingly, despite greater attenuation of stenotic kidney microvascular damage, renal injury, and fibrosis, delivery of MSC-derived EVs achieved similar improvements in renal hemodynamics and function compared to PTRA (Table 2). These observations suggest that PTRA and EVs activate different mechanisms to protect the post-stenotic kidney. In fact, and RBF and GFR exceeded Control levels, likely due to obesity-related hyperfiltration in RVD groups. EVs exerted greater attenuation of major contributors to renal disease progression, such as microvascular rarefaction, inflammation, oxidative stress, fibrosis [49], ultimately improving stenotic kidney function. Contrarily, successful restoration of renal artery patency by PTRA might have improved RBF and GFR by increasing perfusion pressure and downregulating the intra-renal RAAS, disclosed by decreased RV renin levels. Previous studies in two-kidney, one-clip (2K1C) Goldblatt rats demonstrated that the RAAS plays a key role in the development and maintenance of hypertension [50], and contributes to renal injury and fibrosis [51]. Thus, modulation of RAAS by PTRA could have contributed not only to decrease blood pressure, but also to preserve renal function in the stenotic kidney. Contrarily, systemic plasma renin activity remained unchanged in our model, as typical for a chronic phase of RVD [52].

Taken together, these observations suggest that at least short-term improvements in RBF, GFR, and serum creatinine can be achieved with modest decreases in fibrosis and preservation of the intrarenal microvasculature, and without completely switching off renal pathogenic mechanisms. Yet, our results position EVs as a promising therapeutic intervention to preserve the stenotic kidney in RVD. Indeed, a phase II/III pilot study demonstrated that MSC-derived EV therapy is safe and can ameliorate renal inflammation and improve function in patients with CKD [53]. In addition, novel methods for selective targeting injured kidneys, such as conjugation with antibodies directed against kidney injury molecule (KIM)-1, may enhance MSC and EV retention in the ischemic kidney and their abolish the need for intra-renal injections, thus decreasing the invasiveness of this approach [53]. On the other hand, treatment with EVs did not decrease blood pressure, warranting adjuvant anti-hypertensive therapy. Therefore, further studies are needed to confirm their safety and long-term effects, and to select RVD patients likely to benefit from MSC-derived EV therapy.

Our study is limited by the use of relatively young animals and the short duration of the disease relative to humans. Therefore, PTRA was more successful in restoring blood pressure and renal function in our pigs than typically achieved in humans [5]. However, our swine model recapitulates the main features of human RVD, serving as a robust platform to compare the efficacy of PTRA and MSC-derived EVs to preserve the structure and function of the stenotic kidney. Four weeks after intrarenal injection, EVs were detected in post-stenotic kidney sections and co-localized with tubular cells and endothelial cells, but the mechanisms implicated in EV engraftment remain to be determined and explored in future studies. Furthermore, the duration of their benefit and possible need for repeated injections need to be determined.

## 5. Conclusions

In summary, our study shows that a single intra-renal delivery of MSC-derived EVs elicited better preservation of the stenotic kidney microvasculature and attenuation of renal injury and fibrosis compared to renal revascularization, possibly partly attributed to their cargo of vasculo-protective genes. However, both strategies similarly improved renal hemodynamics and function, suggesting that at least short-term improvements in RBF and GFR could be achieved with modest preservation of the intrarenal parenchymal damage. Therefore, our observations bear relevance and may shed light on the specific processes and mechanisms implicated in improvement of post-stenotic kidney function in experimental RVD. Nevertheless, future studies are needed to compare the effectiveness of EVs and PTRA to improve renal function in patients with RVD.

## Figures and Tables

**Table 1:** Systemic characteristics and single-kidney function in study groups at the end of the study (*n* = 6 each).

	Control	RVD + Sham	RVD + PTRA	RVD + EVs
Body weight (kg)	72.3± 5.9	92.2 ± 6.7 *	84.9 ± 11.1 *	86.7 ± 6.3 *
Degree of stenosis (%)	0.0 ± 0.0	82.5 ± 10.4 *	0.0 ± 0.0†	75.5 ± 6.9 *^,‡^
SBP (mmHg)	129.2 ± 6.5	177.0 ± 9.9 *	156.2± 16.5 *^,†^	176.0 ± 10.5 *^,‡^
DBP (mmHg)	81.7 ± 3.7	116.0 ± 16.3 *	97.5 ± 9.9 *^,†^	112.2 ± 8.3 *^,‡^
MAP (mmHg)	97.5 ± 3.6	136.3 ± 12.7 *	117.1 ± 11.3 *^,†^	133.4 ± 7.4 *^,‡^
8-isoprostane (pg/mL)	78.4 (75.4–95.3)	306.8 (243.6–426.9) *	356.6 (255.5–441.4) *	111.0 (95.2–150.6) *^,†,‡^
RV renin (ng/mL)	175.1 ± 11.8	287.1 ± 22.5 *	249.2 ± 15.0 *^,†^	279.7 ± 23.8 *^,‡^
PRA (ng/mL/h)	0.15 ± 0.13	0.17 ± 0.07	0.16 ± 0.05	0.18 ± 0.11
Serum creatinine (mg/dL)	1.22 (1.18–1.23)	1.93 (1.89–2.02) *	1.67 (1.53–1.87) *^,†^	1.62 (1.54–1.77) *^,†^
RBF (mL/min/kg)	7.0 ± 0.6	6.3 ± 0.8 *	9.1 ± 1.7 *^,†^	8.8 ± 0.4 *^,†^
GFR (mL/min/kg)	1.1 ± 0.1	1.0 ± 0.1 *	1.5 ± 0.2 *^,†^	1.5 ± 0.1 *^,†^

RVD: Renovascular disease, SBP: Systolic blood pressure; DBP: Diastolic blood pressure; MAP: Mean arterial pressure, RV: Renal vein; PRA: Plasma renin activity, RBF: Renal blood flow, GFR: Glomerular filtration rate. * *p* < 0.05 vs. Control; ^†^ *p* < 0.05 vs. RVD + Sham; ^‡^ *p* < 0.05 vs. RVD + PTRA.

**Table 2:** Effects of PTRA and MSC-derived EVs on blood pressure, stenotic kidney hemodynamics, and renal injury pathways in swine RVD.

Intervention	RVD + PTRA	RVD + EVs
Blood pressure	↓	=
RBF	↑	↑
GFR	↑	↑
Serum creatinine	↓	↓
Microvascular density	↑	↑↑
Microvascular maturity	↑	↑↑
Peritubular capillary density	↑	↑↑
Renal oxidative stress	=	↓
Renal Inflammation	=	↓
Tubular injury	↓	↓↓
Tubulo-interstitial fibrosis	↓	↓↓
Glomerulosclerosis	=	↓

RBF: renal blood flow, GFR: glomerular filtration rate.

## Data Availability

The pig mRNA data are available online at: https://figshare.com/articles/dataset/Pig_mRNAseq_MSC_vs_EVs_Vasculoprotective_genes/14125877.

## Supplementary Materials

The pig mRNA data are available online at: https://figshare.com/articles/dataset/Pig_mRNAseq_MSC_vs_EVs_Vasculoprotective_genes/14125877.
